# Action goals and the praxis network: an fMRI study

**DOI:** 10.1007/s00429-022-02520-y

**Published:** 2022-06-22

**Authors:** Bartosz Michalowski, Mikolaj Buchwald, Michal Klichowski, Maciej Ras, Gregory Kroliczak

**Affiliations:** 1 Action and Cognition Laboratory, Faculty of Psychology and Cognitive Science, Adam Mickiewicz University, Wydział Psychologii i Kognitywistyki UAM, ul. Szamarzewskiego 89, 60-568 Poznan, Poland; 2 Learning Laboratory, Faculty of Educational Studies, Adam Mickiewicz University, Poznan, Poland

**Keywords:** Affordances, Grasp planning, Hand–tool interactions, Motor cognition, Multi-voxel pattern analysis, Tool grasping

## Abstract

The praxis representation network (PRN) of the left cerebral hemisphere is typically linked to the control of functional interactions with familiar tools. Surprisingly, little is known about the PRN engagement in planning and execution of tool-directed actions motivated by *non-functional* but purposeful action goals. Here we used functional neuroimaging to perform both univariate and multi-voxel pattern analyses (MVPA) in 20 right-handed participants who planned and later executed, with their dominant and non-dominant hands, disparate grasps of tools for different goals, including: (1) planning simple vs. demanding functional grasps of conveniently vs. inconveniently oriented tools with an intention to immediately use them, (2) planning simple—but *non-functional*—grasps of inconveniently oriented tools with a goal to pass them to a different person, (3) planning reaching movements directed at such tools with an intention to move/push them with the back of the hand, and (4) pantomimed execution of the earlier planned tasks. While PRN contributed to the studied interactions with tools, the engagement of its critical nodes, and/or complementary right hemisphere processing, was differently modulated by task type. E.g., planning non-functional/structural grasp-to-pass movements of inconveniently oriented tools, regardless of the hand, invoked the left parietal and prefrontal nodes significantly more than simple, non-demanding functional grasps. MVPA corroborated decoding capabilities of critical PRN areas and some of their right hemisphere counterparts. Our findings shed new lights on how performance of disparate action goals influences the extraction of object affordances, and how or to what extent it modulates the neural activity within the parieto-frontal brain networks.

## Introduction

Using tools in accordance with their functions is but one of numerous ways in which we interact with manipulable objects. After all, we often grasp a tool and hand it to another person or simply displace an object as a transient obstacle. Performing such disparate tasks requires that an appropriate motor plan complies both with constraints imposed by the ultimate action goal (e.g., an intention of using vs. passing or moving a tool) and the current object’s characteristics, such as the orientation of its handle (Valyear et al. 2011). Yet, even if the latter requires at first an uncomfortable or biomechanically awkward hand rotation prior to a goal-relevant grasp, it is usually preferred over the posture that is initially comfortable but later requires quite complex adjustments to complete the intended action (Rosenbaum and Jorgensen 1992; Seegelke et al. 2013). Surprisingly, although it has been long suggested that differently motivated hand–object interactions may be sub-served by functionally and neuro-anatomically distinct networks (e.g., Johnson and Grafton 2003; Daprati and Sirigu 2006; Grafton and Hamilton 2007; Vingerhoets et al. 2009; Buxbaum and Kalenine 2010; Binkofski and Buxbaum 2013), very little is still known about the neural underpinnings of the planning and execution of such disparate actions involving tools (Garcea and Buxbaum 2019).

Up until now, most functional magnetic resonance imaging (fMRI) reports concerning tool-directed manual skills have focused primarily on functional interactions with common tools (Hermsdorfer et al. 2007; Kroliczak and Frey 2009; Vingerhoets et al. 2011; Valyear et al. 2012; Chen et al. 2016; Przybylski and Kroliczak 2017; see also Brandi et al. 2014). When actions motivated by disparate goals were considered, they usually involved simple non-functional objects, such as wooden or plastic blocks, with either simple or irregular shapes (Króliczak et al. 2008; Cavina-Pratesi et al. 2010; Monaco et al. 2011; Gallivan et al. 2013; Marangon et al. 2016). Conversely, when common tools or objects and different actions directed toward them were investigated, it was done primarily in the context of hand posture/action recognition (Buxbaum et al. 2006; Handjaras et al. 2015), action observation (Platonov and Orban 2017; Orban et al. 2019; Urgen and Orban 2021) or imagining of the goal-appropriate hand/finger postures (Vingerhoets et al. 2009, 2013). To the best of our knowledge, the only two studies thus far (Garcea and Buxbaum 2019; Malfatti and Turella 2021), which successfully addressed some differences in neural representations of pantomimed performance of tool-related actions with distinct goals, utilized functional connectivity modeling, and multi-voxel pattern analysis (MVPA) to show consistent signal modulations and their directions in tool/action processing streams, or their decoding capabilities of the goal or general use components of such actions. In our project, we asked a more fundamental question and examined the modulations of neural activity contingent on performance of disparate functional and non-functional grasps of tools, involving the requisite neural computations that must precede the ones for their effective use or other types of handling. To this end, we first utilized univariate fMRI to measure blood-oxygen-level-dependent (BOLD) signal changes associated with planning and subsequent execution of pantomimed grasping of tools with a view to passing them to a different person, performance of demanding or easy functional grasps of tools to effectively use them later on, *versus* reaching actions to move, or push such objects as potential obstacles. As such, all the studied main tasks—grasp types, directed at common tools or utensils, were associated with different goals in mind. Subsequently, we also applied MVPA to identify areas effectively discriminating between the examined action classes.

Based on earlier research, we hypothesized that compared to the control *reach-and-move* (RAM) task, both planning functional grasps, i.e., the *grasp-to-use* (GTU) task, and non-functional or structural grasps, i.e., the *grasp-to-pass* (GTP) task, would involve the left-lateralized praxis representation network (PRN, Frey 2008; Przybylski and Kroliczak 2017; Rossi et al. 2018; cf. Sulpizio et al. 2020). We also predicted that a subset of areas within PRN, located in the inferior parietal, precentral, and lateral prefrontal cortices would be invoked more for planning functional, rather than non-functional/structural, grasps of tools (Buxbaum et al. 2006; Kroliczak and Frey 2009; Vingerhoets et al. 2009; Brandi et al. 2014; Garcea and Buxbaum 2019), as well as efficient decoding of their disparate classes (Malfatti and Turella 2021). Finally, we expected that the left-lateralized PRN activity associated with planning functional, vs. structural, grasps would be similar, regardless of the tested hand (Przybylski and Kroliczak 2017; Buchwald et al. 2018).

## Materials and methods

### Participants

Twenty healthy adult individuals (age range = 20–29, mean age = 24.7, 10 women) with no history of neurological or psychiatric disorders participated in two fMRI sessions. All participants had normal or corrected-to-normal visual acuity, and were strongly right-handed, as determined by the revised version of the Edinburgh Handedness Inventory (mean Laterality Quotient = 96.6, *SD* = 9.2; Oldfield 1971; Dragovic 2004). Prior to testing, an informed written consent was obtained from each volunteer. At study conclusion, participants were compensated financially for their time and efforts, and were debriefed. The study was approved by the Bioethics Committee at the Poznań University of Medical Sciences and was carried out in accordance with the principles of the 2013 WMA Declaration of Helsinki.

### Stimuli

**Fig. 1:**
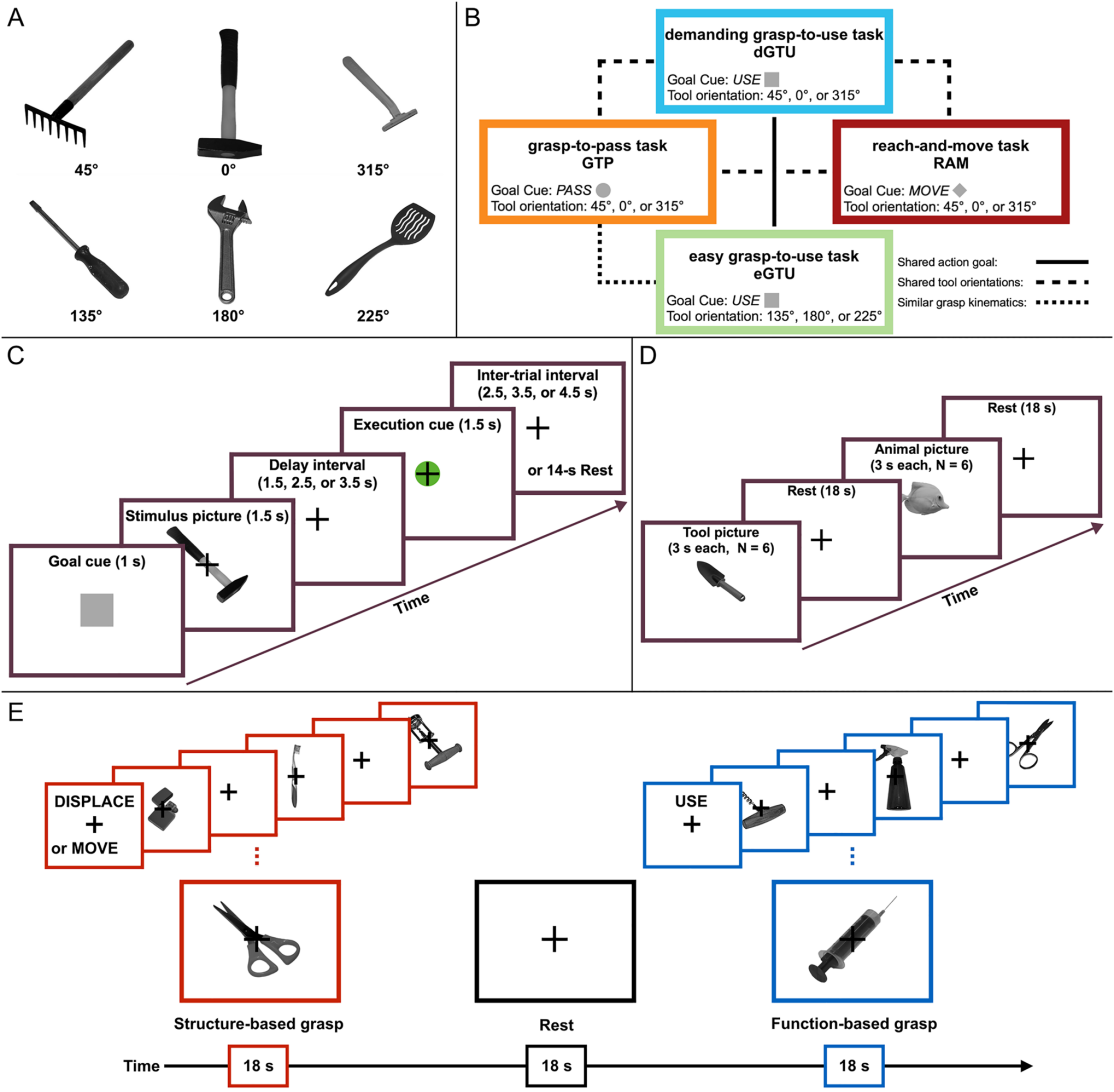
Stimuli, conditions and trial structures. **A** Examples of stimuli used in the main study. Top panel: tools presented at 45, 0, and 315 degrees. Bottom panel: tools presented at 135, 180, and 225 degrees. **B** Four study conditions resulting from the combination of three different action goals (indicated by goal cues) and two sets of stimulus orientations. **C** Trial structure and timing of the main experiment, using an event-related design. **D** Trial structure and timing of the visual tool use localizer task with a block design. **E** Trial structure and timing of the background study on structure-based (i.e., grasping to displace or move) vs. function-based pantomimed grasping of tools, utilizing a block design

The stimuli consisted of 72 high-resolution, greyscale photos of 12 different graspable common objects, such as mechanical tools, garden implements, office or kitchen utensils and personal care items. Depending on their real sizes, half of the stimuli would require a precision grip, and the other half a power grip to manually handle them. Each object was photographed with a Sony DSC-H50 digital camera on a white background, in six different orientations (i.e., 0, 45, 135, 180, 225, and 315 degrees), and presented during the experiment in its foreshortened view, emulating the perspective of a person standing by the table on which the tool was placed. Examples of the objects used in this study are presented in Fig. 1A, and a list of all the objects can be found in the Appendix.

### Setup and apparatus

Participants were positioned head first and supine in the magnet bore with both their arms laid alongside the body. A pair of MRI-compatible two-button response devices (Lumina LU400- PAIR manufactured by Cedrus, San Pedro, CA, USA) was attached to the scanner bed with Velcro stripes, one response pad on each side of the body. To reduce scanner noise, participants were provided with earplugs and headphones. Head movements were restricted by fixing the head in place with foam cushions. Stimulus presentation and response recording were controlled by SuperLab ver. 4.5.3 software (Cedrus, San Pedro, CA, USA) digitally synchronized with the MRI scanner. The stimuli were projected onto a 32-inch NordicNeuroLab LCD monitor (NordicNeuroLab, Bergen, Norway) positioned at the back of the scanner and viewed via a tilted mirror attached to the head coil.

### Procedure

#### Main experiment

BOLD fMRI signal was measured while participants planned and executed tool-oriented pantomimed actions in an event-related design (e.g., Kroliczak and Frey 2009; Przybylski and Kroliczak 2017). All study participants completed two separate sessions on consecutive days, using their dominant right and non-dominant left hands. The order of the tested hands was counterbalanced across participants.

The main experiment consisted of 6 functional runs with 24 trials each. At the beginning of each run, participants were asked to press one of the buttons of the response pad with the index finger of the tested hand, and maintain the pressing throughout the whole run, except for the pantomimed execution of the planned actions. This manipulation made it possible to control the exact moment of movement onset and thereby to omit in analyses the trials wherein participants erroneously initiated the movement during the planning phase. Every trial began with a variable interval of 0, 0.25, 0.5, or 0.75 s, so that stimulus onset occurred at a variable delay relative to the onset of the functional volume acquisition (Miezin et al. 2000), followed by a 1-s *Goal Cue* presented centrally in the form of one of three geometrical shapes of different colors, which denoted an action goal for a given trial, resulting in three main tasks. In (1) the GTU (grasp-to-use) task, indicated by a tan square, the to-be-seen object should be grasped in a way that would allow for its immediate use, without any further adjustment of the hand/wrist posture; there were two versions of the GTU task, a demanding, and an easy one, contingent on object rotation; in (2) the GTP (grasp-to-pass) task, indicated by a pink circle, the object should be grasped in a simple or convenient way—i.e., without unnecessary hand rotation, with an intention to pass it to another person positioned in front of the participant; in (3) the RAM (reach-to-move) task, indicated by a blue diamond (rhombus), the object should be reached and simply moved (pushed) with the back of the hand. Subsequently, an image of the target object was presented centrally for 1.5 s. The main characteristics of these tasks are illustrated in Fig. 1B. Participants were instructed to prepare to pantomime the grasp identified by both the intention cue and the stimulus image as soon as the image appeared and throughout the subsequent delay period of variable duration (1.5, 2.5, or 3.5 s). Next, the *execution cue* (a bright green circle, with an embedded fixation cross, presented for 1.5 s in the middle of the screen) prompted participants to release the depressed button and to simulate a pre-planned functional or non-functional—but structurally appropriate—grasping action or reaching movement. Because of the supine position in the scanner, all tasks were performed without visual feedback. Participants were instructed to use only the fingers, hand and forearm, with the upper arm remaining still. At the offset of the execution cue participants returned their hands to the starting position, and resumed pressing the button with the index finger. Each trial concluded with a variable inter-trial interval (ITI) of 2.5, 3.5, or 4.5 s, and, if necessary, an additional short period for synchronization with the scanner trigger. In each run, 4 additional 9.5-s rest intervals were introduced pseudorandomly at the end of the trials with the longest ITIs, providing four 14-s periods serving as resting baseline. Trial structure and timing are shown in Fig. 1C. During each run, participants were instructed to fixate on a centrally presented cross. Manual performance was monitored by the experimenter. Trials in which the executed action did not match the specified action goal, the presented target object, and/or its orientation (e.g., functional grasp performed instead of reaching and moving the object, precision grip performed instead of a power grip, no wrist rotation performed in the case when a substantial rotation was necessary) were omitted from analyses. Yet, only 66 out of 2880 trials (2.9%) completed with the right hand, and 87 out of 2880 trials (3.8%) completed with the left hand were not included in further analyses due to errors in action execution or action timing.

Whether, from the perspective of a participant, the task was demanding or easy depended on specific stimulus orientation. Therefore, in fact, there were four testing conditions distinguished based on a goal cue and/or a subset of orientations at which target objects were presented (see Fig. 1B):

1. In the demanding GTU task (dGTU), the *use* cue followed by an object presented in one of three different orientations — 0°, 45°, or 315° — required the inclusion of a substantial wrist rotation in the grasp plan.
2. In the easy GTU task (eGTU), the *use* cue followed by an object presented in one of three different orientations — 135°, 180°, or 215° — required the inclusion of a minor (if any) wrist rotation in the grasp plan.
3. In the GTP task, the *pass* cue followed by an object presented in one of three disparate orientations — 0°, 45°, or 315° — required a simple structural grasp plan.
4. In the RAM task, the *move* cue followed by an object presented again in one of three disparate orientations — 0°, 45°, or 315° — required a pushing movement with the back of the wrist.

It is of note that trials in the dGTU and eGTU tasks shared the action goal. Trials in the dGTU and GTP tasks shared tool orientations. Finally, trials in the eGTU and GTP tasks shared similar grasp kinematics.

Throughout the experiment, each of the 12 target objects was presented 3 times in every condition, each time in a different orientation. Such object orientation–intention/goal triplets were distributed pseudorandomly across 6 complementary orders of trials in a counterbalanced manner so that every order consisted of 24 trials (6 trials in each condition) presented in a pseudorandom sequence. Targets from each condition had an equal likelihood of being followed by either of the 3 delay intervals and ITIs. The sequence of presentation of the 6 orders was pseudorandomized across participants and testing sessions so that each participant was assigned each order twice, once during each testing session, and every assigned sequence was unique within the whole study.

One day prior to the first fMRI testing session, each participant took part in a training session. First, participants were familiarized with the scanning procedures, completed a pre-scan MRI-safety questionnaire and a revised Edinburgh Handedness Inventory. Subsequently, each participant completed a minimum of two series of 24 training trials of our experimental paradigm, presented on a computer screen and administered separately for each hand. Stimuli used during the training session were different from the stimuli presented in the main experiment. For every participant, the order in which the hands were trained was the same as the order in which they were subsequently tested during the fMRI sessions. It was strongly emphasized that all the manual movements should be performed precisely but in a calm manner, and the head motion during the scanning must be eschewed. All participants performed faultlessly before being advanced to the testing sessions.

#### Additional localizer scans

##### Tool use pantomimes

All the 20 participants were also tested twice (once per session) in a functional *Tool Use Localizer* (TUL) which served to identify brain areas associated with pantomiming the functional use of familiar tools. There were eight 18-s task blocks in the TUL scans, four 18-s blocks of pantomimed tool use (i.e., performance of tool use gestures) in response to pictures of tools displayed for 3 s each (6 stimuli in each block) and four 18-s blocks of more abstract hand and finger movements in response to pictures of animals presented for 3 s each (6 stimuli in each block). In the latter case, the required manual movements depended on the way the presented animal usually moves, and there were three possible responses: (1) moving fingers back and forth and gently extending the arm forward for animals that walk or run on their legs, (2) waving the vertically oriented hand from side to side and moving the arm forward for animals that swim, and (3) waving the horizontally oriented hand up and down and moving the arm forward for animals that fly. These movements were presented and rehearsed during the training session and immediately prior to each of the testing sessions. In both tasks, participants were instructed to start the movement as soon as a new picture (a tool or an animal) appeared and to continue as long as the picture was displayed. All actions were performed with the same hand (dominant right or non-dominant left) that was tested in the main experiment during the same fMRI session. Additional four 18-s blocks of rest periods were introduced pseudorandomly between task blocks. A black cross remained in the center of the screen during the whole functional run and served as a fixation point. A schematic diagram of the block structure and timing is displayed in Fig. 1D. Two different pseudorandom orders of task and rest blocks were prepared and administered to each participant. The assignment of the two orders was counterbalanced across the tested hands. A list of tools used in TUL can be found in the Appendix. The objects, or their exemplars, are different from tools used in the main study to avoid any confusion as to the required pantomimes (i.e., simulated grasp vs. use).

##### Grasp-to-displace pantomimes and their functional counterparts

The grasp-to-displace (GTD) localizer task served to identify brain areas associated with processing of structural tool information for grasping a tool to displace it or put it aside (a reference, structure-based condition). It was contrasted with processing of functional tool information for grasping with a view to using a tool (a function-based condition), involving the retrieval of conceptual knowledge and/or selection of functional hand postures typically associated with common tools. In the latter case, i.e., the control grasp-to-use (cGTU) condition, participants were instructed to pantomime grasping the presented tool with an intention to subsequently use it according to its function. Notably, in contrast to structural grasp-to-pass pantomimes performed in the main experiment, structure-based grasps in the GTD task did not require our participants to consider the more distant context of the action, i.e., the information about the recipient positioned in front and to whom the tool is handed in. There were twelve 18-s task blocks in the GTD localizer scans, six blocks of structure-based grasps and six blocks of function-based grasps in response to pictures of tools displayed for 1.5 s each (6 stimuli in each block, 1.5-s inter-stimulus interval, ISI). The stimuli for the GTD and cGTU tasks were selected to induce “action conflict” whereby different hand pre-shaping and postures are required for moving or for using the same object (Buxbaum et al. 2006; Watson and Buxbaum 2015). A list of tools used in the grasp-to-displace localizer can be found in the Appendix. Six different tools were used, three different exemplars of each tool, with each exemplar presented at three different orientations: 135°, 180°, and 225°. Each task block began with an instructional cue—DISPLACE for the GTD condition, and USE for the cGTU condition, displayed for 1 s above the central fixation cross, which remained on the screen during the whole functional run. Participants were instructed they should start to pantomime the task-appropriate grasp as soon as a new stimulus picture appeared on the screen, maintain the final hand posture as long as the stimulus was displayed, and return to the resting position during ISIs.

All actions were performed with the same hand (dominant right or non-dominant left) that was tested in the main experiment during the same fMRI session. Additional six 18-s blocks of rest periods were introduced pseudorandomly between task blocks. A schematic diagram of the block structure and timing is displayed in Fig. 1E. As in the TUL scans, two different pseudorandom orders of task and rest blocks were prepared and administered to each participant. The assignment of the two orders was counterbalanced across the tested hands.

The results obtained in these additional localizer scans turned out to be critical for understanding the outcomes from the main experiment. Therefore, in the Results section they will be reported in the reversed order first.

### Data acquisition

All scanning was performed in the Rehasport Clinic (Poznan, Poland) on a 3-Tesla Siemens MAGNETOM Spectra MRI scanner (Siemens Healthcare, Germany) using a 16-channel head coil for radio frequency transmission and signal reception. The BOLD echoplanar (EPI) images were collected using a T2*-weighted gradient echo sequence with the following parameters: time to repetition (TR) = 2000 ms, time to echo (TE) = 30 ms, flip angle (FA) = 90°, voxel matrix = 58 × 64, Field of View (FoV) = 181.25 × 200 mm, 35 axial slices with in-plane resolution of 3.125 × 3.125 mm and slice thickness of 3.1 mm. Standard T1-weighted structural images were acquired using a 3D magnetization-prepared rapid gradient echo (MP-RAGE) pulse sequence: TR = 2300 ms, TE = 3.33 ms, inversion time (TI) = 900 ms, FA = 9°, voxel matrix = 240 × 256, FoV = 240 × 256 mm, 176 contiguous sagittal slices, 1.0-mm isotropic voxels. To improve the accuracy of functional-to-anatomical data co-registration, fast spin echo T2-weighted structural images were also collected: TR = 3200 ms, TE = 417 ms, FA = 120°, voxel matrix = 256 × 256, FoV = 256 × 256 mm, 192 contiguous sagittal slices, 1.0-mm isotropic voxels. Raw image data were converted to NIfTI-1 format using MRI-Convert software (http://lcni.uoregon.edu/downloads/mriconvert).

### Structural and functional image analyses, including MVPA

All structural and functional images were analyzed using FSL (FMRIB’s Software Library, http://fsl.fmrib.ox.ac.uk/fsl/) version 5.0.7 or later (Jenkinson et al. 2012). First, two high-resolution T1-weighted structural images acquired for each participant were averaged using FLIRT (Jenkinson and Smith 2001; Jenkinson et al. 2002) and subjected to the removal of non-brain tissue using BET (Smith 2002). Subsequently, functional images were analyzed with FEAT (FSL’s FMRI Expert Analysis Tool) version 6.0. Preprocessing procedures included: the removal of non-brain tissue using BET; motion correction using MCFLIRT; spatial smoothing using a Gaussian kernel of full width at half-maximum (FWHM) = 6.2 mm; grand mean intensity normalization of the whole 4D data set by a single scaling factor; and a high-pass temporal filtering cutoff of 50 s (σ = 25 s). Before statistical analyses, auto-correlation in the data was corrected using a pre-whitening procedure (Woolrich et al. 2001). Hemodynamic responses were modeled using a double-gamma function. For spatial normalization, functional (EPI) images were first co-registered to the T2-weighted anatomical image with 6 degrees of freedom (DOF). Next, T2- and T1-weighted (MP-RAGE) images were aligned with the use of Boundary-Based Registration (Greve and Fischl 2009). Finally, registration of the T1-weighted image to the standard Montreal Neurological Institute (MNI-152) 2-mm template brain was performed using 12 DOF.

For a given participant, each fMRI run was analyzed separately at the first level. Planning-related activity in each condition was modeled as the 3-s period beginning with the onset of the target stimulus (presented for 1.5 s) and lasting throughout the end of the shortest delay interval (1.5 s). Execution-related activity was modeled as the 1.5-s period during which the execution cue was displayed. Resting baseline was modeled as the 14-s period starting with the offset of the execution cue through the longest ITI and additional 9.5-s interval. In the localizer scans, the durations of the whole blocks were modeled, and the outcomes collapsed across both hands. Within-subjects analyses were performed using a fixed effects model implemented in the FSL’s FEAT. Group analyses were performed using FLAME (FMRIB’s Local Analysis of Mixed Effects) Stage 1 (Beckmann et al. 2003) to model and estimate random-effects components of mixed-effects variance. The resulting Z (Gaussianized t/F) statistic images were thresholded with a cluster-forming threshold of Z > 3.1 and a family-wise error rate (FWER) controlled at alpha = 0.05 (Eklund et al. 2016). For the TUL analysis at the group level, a more conservative threshold of Z > 4.0 (i.e., half of the maximum Z for its control task) was used to reveal the asymmetric — strongly lateralized — contributions of the two hemispheres to the two studied tasks.

Because the univariate analysis for the main experiment utilized an event-related design, in the MVPA analysis of these data, we first calculated parameter estimates (PEs) for each trial. Namely, we created a general linear model (GLM) separately for each trial with the use of the “Least Squares – Separate approach” (LS-S, see: Mumford et al. 2012). Within each run, each model was created for every trial with two Explanatory Variables (EVs): the first was the Trial of Interest (TOI), and the second consisted of all the remaining, other trials (OT). Specifically, because there were 6 trials per condition during one functional run (i.e., 12 trials for two conditions in a run), 12 GLMs were created to obtain one PE for each trial (12 × 6 runs, which gives 72 matrices of *beta* values altogether). Prior to the MVPA, PEs were not normalized. Two pairs of conditions were initially considered: (1) decoding the planning phase of the dGTU task, as compared with the GTP task; (2) planning phase of the eGTU task, as compared with the GTP task.

Because the number of samples in each of the decoded class was balanced, the *leave-one-trial-out* cross-validation method was evaluated using the accuracy metric. As there were 6 trials per condition for one run, and 6 runs in total, each fold of the validation consisted of 71 PEs (6 × 2 × 6–1; trials × conditions × runs—one trial for validation) for training the classifier and the 1 left-out PEs to test the accuracy of the classification. This operation was performed 72 times and the classification accuracies were averaged with the arithmetic mean. Thus, per each hand, for each participant, the single accuracy score was obtained in a range of 0.0 to 1.0 (i.e., it could vary between 0 and 100%). SVM (support vector machine) model was used to perform the MVPA classification (linear kernel, *C* parameter fixed at 1.0), as implemented in scikit-learn Python module (Abraham et al. 2014; http://scikit-learn.org/stable), using the nilearn module (http://nilearn.github.io).

Regardless of the analysis type (univariate, multivariate), clusters with significant brain activity were localized by projecting and visualizing the obtained volumes or patterns with the use of the Connectome Workbench v1.4.2 (Marcus et al. 2011; Glasser et al. 2016a). With this software, group statistical imaging maps are overlaid, here — with the use of trilinear interpolation, onto inflated mid-thickness and flat surfaces. These maps were additionally demarcated with borders of critical functional areas, based on the multi-modal parcellation schemes. The neuroanatomical labels applied throughout this report are also taken from the “connectome workbench” atlas (Glasser et al. 2016b). For the roots of the terminology used, see also a publication by Triarhou (2007).

### Region of interest (ROI) analyses

The principal goal of the ROI analyses was to investigate the exact patterns of activity related to planning different interactions with tools within areas typically implicated in the preparation and/or execution of complex manual actions involving tools (Kroliczak and Frey 2009; Jacobs et al. 2010; Marangon et al. 2011; Brandi et al. 2014; Vingerhoets and Clauwaert 2015). Seven left hemisphere regions were chosen as ROIs: the rostral middle frontal gyrus (rMFG, here: area 46), ventral premotor cortex (PMv, 6r/IFJp), the supplementary motor/dorsal premotor cortex (SM/PMd, 6mp, 6d), anterior intraparietal cortex (aIPS, AIP/area 2), anterior supramarginal gyrus (aSMG, PF/PFt/PFop), caudal superior parietal lobule (cSPL, VIP/MIP/7PL), and caudal middle temporal gyrus (cMTG, parcels PH/PHT in the connectome workbench nomenclature). All ROIs were defined at the group level as spheres of 5-mm radius centered on maximally activated voxels from clusters involved in pantomiming the use of familiar tools (vs. simulated animal movements) in the Tool Use Localizer task, irrespective of the hand performing the task. MNI coordinates of the peak voxels and their Z values are given in Table 1. Their locations were initially established with help from the Juelich Histological, and Harvard–Oxford probabilistic atlases implemented in the FSL, and subsequently also projected to the connectome workbench atlas.

**Table 1:** Regions of interest used in the current study

Left hemisphere region	MNI coordinates			Peak *Z* value	Main effect of hand	Main effect of phase	Main effect of task	Significant interactions
	*x*	*y*	*z*					
Rostral middle frontal gyrus (rMFG, 46)	– 40	40	30	4.59	*p* = 0.19	*p* = 0.80	*p* < 0.001, PES = 0.36	(P × T): *p* < 0.001, PES = 0.42 (H × P): *p* < 0.01, PES = 0.31
Ventral premotor cortex (PMv, 6r/IFJp)	– 46	4	24	6.01	*p* = 0.31	*p* < 0.01, PES = 0.36	*p* < 0.001, PES = 0.40	(P × T): *p* < 0.001, PES = 0.31
Supplementary motor / dorsal premotor cortex (SM/PMd, 6mp, 6d)	– 20	– 14	68	6.50	*p* < 0.001, PES = 0.69	*p* < 0.001, PES = 0.91	*p* < 0.001, PES = 0.36	(P × T): *p* < 0.001, PES = 0.51
Anterior intraparietal sulcus (aIPS, AIP/area 2)	– 38	– 34	40	6.00	*p* = 0.16	*p* < 0.05, PES = 0.20	*p* < 0.001, PES = 0.57	(P × T): *p* < 0.001, PES = 0.51
Anterior supramarginal gyrus (aSMG, PF/PFt/PFop)	– 62	– 26	34	7.35	*p* = 0.84	*p* < 0.01, PES = 0.35	*p* < 0.001, PES = 0.43	(P × T): *p* < 0.001, PES = 0.41 (H × P): *p* < 0.05, PES = 0.27
Caudal superior parietal lobule (cSPL, VIP/MIP/7PL)	– 22	– 68	58	6.22	*p* = 0.75	*p* = 0.12	*p* < 0.001, PES = 0.47	(P × T): *p* < 0.001, PES = 0.50
Caudal middle temporal gyrus (cMTG, PH/PHT)	– 48	– 64	− 6	6.93	*p* = 0.49	*p* < 0.001, PES = 0.58	*p* < 0.001, PES = 0.43	(P × T): *p* < 0.001, PES = 0.33

ROIs were based on maximally activated voxels from clusters involved in the tool use pantomime task performed in an independent localizer. MNI coordinates of the peak voxels, their *Z* values, and the results of statistical analyses are presented

Note: Hand (H) = right, left; Phase (P) = planning, execution; Task (T) = demanding grasp-to-use (dGTU) task, easy grasp-to-use (eGTU) task, grasp-to-pass (GTP) task, reach-and-move (RAM). PES = partial eta squared

Within each ROI, mean percent signal change relative to the resting baseline was calculated separately for each participant and each condition with the use of FSL’s FEATQuery. The obtained data were submitted to separate repeated-measures Analyses of Variance (rmANOVAs) for each ROI with hand (right, left), phase (planning, execution) and task (demanding *grasp-to-use*, easy *grasp-to-use*, *grasp-to-pass*, *reach-and-move* tasks) as three within-subjects factors. The adopted level of significance was alpha = 0.05, and the required post-hoc tests were Bonferroni-corrected (*p *value corrected for multiple comparisons marked as Bf-*p*).

## Results

### The outcomes from the background localizer scans

#### Pantomimed structure-based vs. function-based grasping

**Fig. 2:**
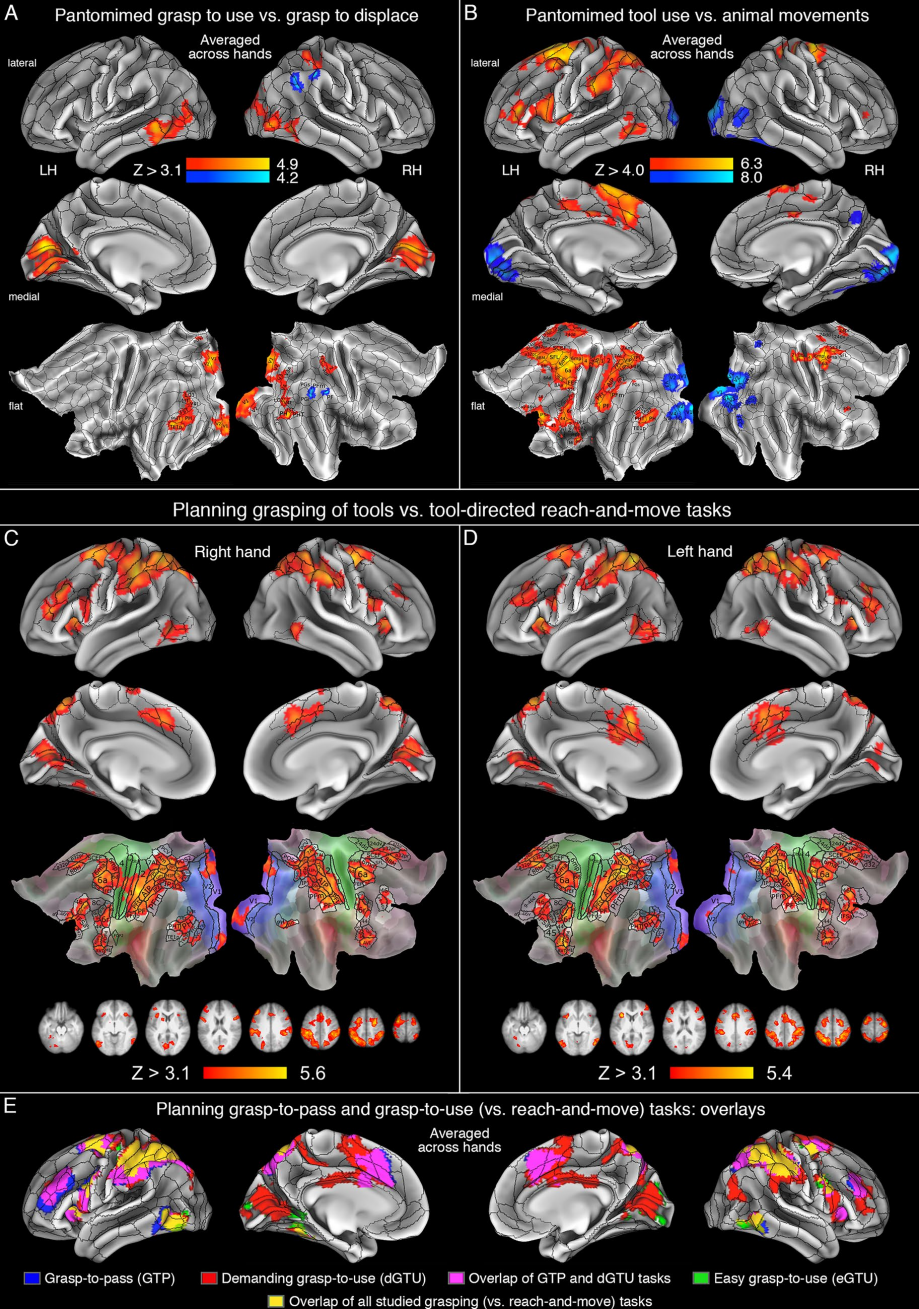
Brain areas showing significant increases of neural activity during critical localizer tasks, and background comparisons from the main experiment, involving the planning of tool-directed grasps compared to the planning of the reach-and-move task. **A** Significantly greater neural activity observed for pantomimed grasp-to-use vs. grasp-to-displace task, shown in warm colors, and its inverse contrast shown in cold colors, collapsed across the dominant right and non-dominant left hand. **B** Neural activity in pantomimed tool use vs. manually simulated animal movements (shown in warm colors), and its inverse contrast (shown in cold colors), collapsed across hands. **C** Planning of tool-directed grasp pantomimes with the right hand. **D** Planning of tool-directed grasp pantomimes with the left hand. Both in (**C**) and (**D**), the obtained neural activity was averaged across three different study conditions, involving difficult grasp-to-use, easy grasp-to-use, and grasp-to-pass tasks. **E** Overlays of neural activity for the three tasks, contrasted separately with the reach-and-move task, but collapsed across the two hands. The obtained clusters, and their most representative slices in panel C and D, were thresholded at least at *Z* > 3.1, and a corrected cluster significance threshold of *p* = 0.05. Volumetric surface renderings were obtained by means of trilinear interpolation, and their projection onto mid-thickness inflated, and flat surfaces of the *connectome workbench* atlas, and subsequently demarcated with borders of multi-modal parcellations implemented in this software. The labels of the involved areas can be found on flat maps, and more detailed descriptions of the obtained effects can be found in the main text

Structure-based (GTD, i.e., grasp-to-displace) vs. function-based (cGTU, i.e., control grasp-to-use) pantomimed grasping movements, collapsed across the tested hands, were associated with just two clusters of significant signal changes in the inferior parietal lobule (IPL) of the right hemisphere. The more anterior one was located at the border of area PF and PFm, and the more posterior one was at the intersection of areas PFm, PGi, and PGs. Given the paucity of greater neural responses for the structure-based (vs. function-based) grasp pantomimes, the obtained clusters are shown in Fig. 2A in cold (dark to light blue) colors.

The inverse contrast of the cGTU and GTD tasks showed significant bilateral engagement of the lateral occipito-temporal cortices (LOTC, including LO3, MT, V4t, FST, PH, PHT through TE1p on the left, and mainly LO2, V4t, FST, PH, and PHT on the right), with somewhat greater extent of neural activity on the left. Moreover, there were significant bilateral contributions from early visual cortices (EVC, V1 through V4 on the right, and V1 and V2 on the left). In the left hemisphere, there was an additional cluster of neural activity in the parieto-occipital sulcus (area POS1), whereas in the right hemisphere an additional cluster was observed at the intersection of areas 7PC, AIP, and area 2. These results are presented in Fig. 2A in warm (red to yellow) colors.

#### Pantomimed tool use vs. simulated animal movements

A direct contrast of tool use pantomimes and manually simulated animal movements showed the expected left hemisphere advantage for the tool use gestures, including areas typically linked to PRN. All the clusters, and their subdivisions disclosed by this contrast are shown in Fig. 2B in warm colors. In addition to bilateral contributions from more superior subdivisions of the sensorimotor areas, significant changes in neural activity were located exclusively in left LOTC (areas PH, PHT, and partly TE1p, which also contributed more to the cGTU task discussed above), IPL (mainly including areas PF, and PFt), the nearby intraparietal sulcus (IPS, e.g., AIP, IP2, LIP and MIP), SPL (extending from 7PC through 7PL), the lateral inferior frontal cortex (IFC, except for bilateral contributions from IFSa, e.g., areas 45, 44, 6r), and the nearby opercular cortex. The contributions from the superior and medial frontal cortices were less asymmetric, and included subdivisions of the supplementary motor area (SMA, e.g., parcels SFL, and SCEF) and cingulate motor regions (e.g., p24pr, and p34pr). Significantly greater right hemisphere contributions to tool use pantomimes were limited to dorsal premotor areas (6a, 6mp, 6ma, extending slightly to SFL and SCEF), small clusters in the cingulate cortex (e.g., p24pr), and IFC (only IFSa).

The inverse contrast of manually simulated animal movements and tool use pantomimes, in addition to bilateral contributions from EVCs (V1–V3, on the left, and V1–V4, on the right, here also extending further to areas V8 and PIT, and even V4t/MT), revealed a relatively large contribution from the right precuneus (7M) and anterior fusiform cortex (FFC). These outcomes are shown in cold colors in Fig. 2B.

### Planning grasping of tools (dGTU, eGTU, GTP) vs. reaching toward tools (RAM) tasks

A comparison of planning all tool-directed grasps, irrespective of action goal and tool orientation, i.e., dGTU, eGTU, and GTP vs. RAM with the right hand revealed a network of parieto-frontal and occipito-temporal areas, including the crucial nodes of PRN. As shown in Fig. 2C, in addition to significant neural activity observed exclusively in the left sensorimotor cortex (including areas 4, 3a, 3b, and 1), most of the remaining clusters of significant changes were bilateral. In SMG this activity included areas PFt, PF, and PFm, as well as PFop exclusively on the left, and in the nearby IPS the significant increases of neural activity extended from AIP through POS2 (including IP2, LIPv, LIPd, VIP, MIP, IPS1, IP1, and DVT, as well as IP0 exclusively on the left). More dorsally, the SPL activity was also bilateral (and included parcels 7PC, 7Am, 7Pm, 7AL, 7PL). In LOTC, the changes in neural activity were observed primarily in cMTG (bilaterally in PHT and PH, but also extending to FST and V4 on the left), and a separate more ventral cluster (involving left FFC and VVC). The bilateral increases observed in early visual cortices were limited only to V1 and V2. In frontal regions, especially in premotor areas, the significant clusters were located bilaterally in PMv (including areas 6r, IFJa, IFJp, PEF, and 44), and the SM/PMd vicinity (including areas 6ma, 6mp, 6d, 6a, i6-8, FEF, extending to area 4). The remaining three clusters were the following: the bilateral antero-ventral insular activity (AVI) extending to the frontal opercular areas (FOP4, FOP5) was largely symmetrical; the lateral prefrontal activity was either bi-hemispheric (in areas 46 and p9-46v), or left-lateralized (in a9-46v, p47, and IFSa parcels); and finally, the medial frontal/prefrontal clusters in the SMA complex (SMA, and preSMA) extended to the cingulate cortex, as well (and involved the following areas: d32, a32pr, p32pr, SCEF, and 8BM, as well as 24dd, and 24dv). This activity was nearly symmetrically invoked in both hemispheres.

The same contrast obtained for tasks performed with the left hand revealed a very similar pattern of results. This time, however, the engagement of the rMFG vicinity was more symmetrical, while the sensorimotor engagement (of areas 4, 3a, 3b, and 1) was almost exclusively right-lateralized. These outcomes are shown in Fig. 2D.

Overlays of neural activity obtained separately for the three main tasks, while contrasted with the control reach-and-move task, are shown in Fig. 2E. Because the patterns of neural activity were very similar for the right and left hand, for simplicity, they were averaged across hands. The planning of the eGTU task invoked the least neural activity, but it overlapped almost entirely with the remaining two tasks. Most of the common activity was bilaterally symmetrical. In the left hemisphere, there was also a substantial overlap of the remaining neural activity associated with the dGTU and GTP tasks. Yet, performance of the dGTU task was associated with greater and/or more widespread activity, especially in the right hemisphere.

### Planning of dGTU vs. GTP and vice versa

**Fig. 3:**
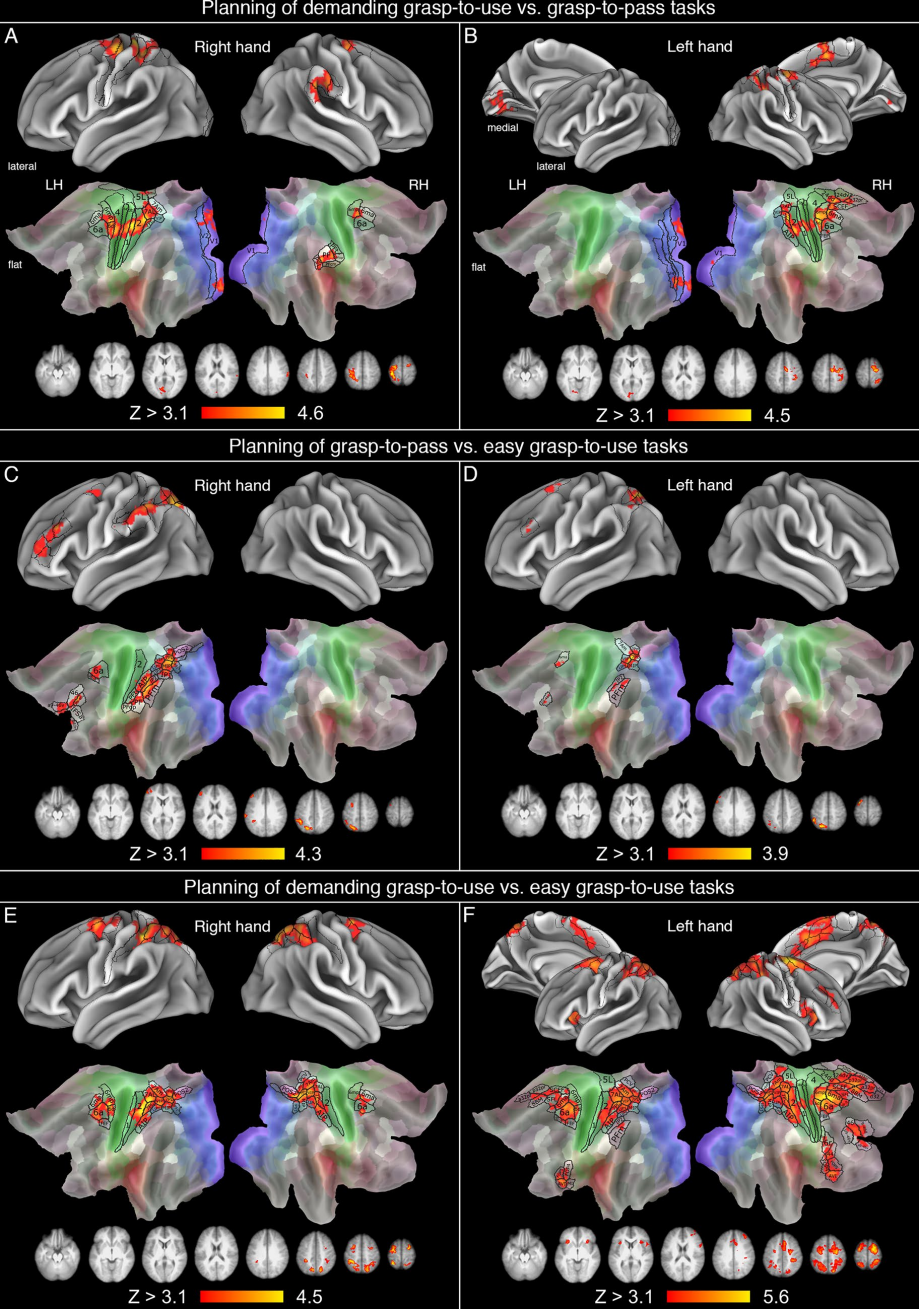
Brain areas showing significant increases of neural activity during planning different tool-directed grasps, contingent on action goal and tool orientation. Brain areas with significantly greater increases for the planning of demanding grasp-to-use, as compared to grasp-to-pass tasks with (**A**) the right hand, and (**B**) the left hand. Areas showing significantly greater increases of neural activity during the planning of grasp-to-pass as compared to easy grasp-to-use tasks with (**C**) the right hand, and (**D**) the left hand. Areas with significantly greater increases of neural activity for the planning of demanding as compared to easy grasp-to-use tasks, with (**E**) the right hand, and (**F**) the left hand

As shown in Fig. 3A, a direct contrast of planning the dGTU vs. GTP task (i.e., the demanding functional, and convenient structural grasp conditions, sharing tool orientations, but having different goals of using or passing, and the associated differences in hand orientation) performed for the right hand revealed significantly increased neural activity in the left primary sensorimotor cortices (4, 3a, 3b, 1, and 2), extending caudally toward the anterior division of SPL (7AL, extending ventrally to 7PC, and dorsally to 5L), and rostrally toward left PMd (6d) and the supplementary motor cortex (mainly 6mp). Notably, the 6mp subdivision was invoked bilaterally. Furthermore, there was a right-lateralized activity restricted to aSMG (namely the rostro-ventral subdivision of PF), and the nearby peri-sylvian area (dubbed PSL). Interestingly, there were also left-lateralized early visual contributions, primarily from V1, but extending both to dorsal and ventral V2. As shown in Fig. 3B, a direct contrast of planning a demanding functional grasp-to-use vs. convenient structural grasp-to-pass performance with the left hand, revealed a similar, but now strongly right-lateralized neural activity, with significant increases observed in the primary sensorimotor cortices (4, 3a, 3b, 1, and 2), also extending caudally toward the anterior division of SPL (primarily 7AL, and 7PC, but also more ventrally to AIP), and rostrally toward PMd (6d) and the supplementary motor cortex (6mp, 6ma, SCEF). It is of note that the right-lateralized engagement was also more medial, and included subdivisions of the cingulate motor area (24dd, 24dv, and the nearby area p32pr). As before, there were also early visual contributions primarily from the left hemisphere, but now extending ventrally from V1 through V4.

The inverse contrasts of the GTP vs. dGTU tasks were, however, empty. Namely, neither for the right nor the left hand was there any advantage, or greater engagement, observed either within or outside of PRN for the planning of convenient structural grasps (to pass), as compared to demanding functional grasps (to use).

### Planning eGTU vs. GTP and vice versa

When planning the eGTU task was contrasted with planning the GTP task for the right hand, none of the areas associated with the praxis network were engaged more by planning easy functional grasps. The only significant increases in neural activity (not shown here) were observed within the bilateral visual cortices (in V1 through V4, on the ventral surfaces), as well as in the nearby parieto-occipital/retrosplenial/posterior cingulate cortex (POS1, v23ab). Consistently with little effects observed for the right hand, the comparison of planning eGTU vs. GTP for the left hand yielded no significant results.

Counter to our hypothesis, the inverse contrasts of planning the GTP and eGTU tasks — accounting for grasp kinematics during structural and easy functional grasps — revealed exclusively left-lateralized engagement of PRN. In the case of tasks performed with the right hand, significant increases in neural activity were detected in rMFG (area 46, p9-46v, a9-46v, and p47r), PMd (6a, i6-8), cSPL/IPS (7PL, 7Pm, MIP, VIP, LIP, IP1, and IPS1), aIPS (AIP, IP2), extending further to SMG (subdivisions PFm, PF, and PFt). These results are shown in Fig. 3C. In the case of tasks performed with the left hand, as shown in Fig. 3D, significant increases in neural activity were observed also in rMFG (but now only in p9-46v), PMd (6ma), cSPL/IPS (7PL, 7Pm, VIP, MIP, LIPv), and finally SMG/IPS (PFm/IP2).

### Planning dGTU vs. eGTU tasks

When planning of the dGTU task was contrasted with planning the eGTU task for the right hand, namely when demanding functional grasps were compared with the easy ones, increased neural activity was observed bilaterally in PMd/SM cortex (6a, 6ma, and 6mp, and exclusively in 6d on the left). There was also a large bilateral posterior cluster, extending from the somatosensory cortex to the parieto-occipital sulcus (involving the following areas: 2, AIP, 7PC, 7AL, 7Am, 7Pm, 7PL, LIPv, VIP, MIP, IPS1, DVT, and POS2). For detailed depiction of this effect, see Fig. 3E. In the case of the left hand, the contrast of dGTU and eGTU revealed even greater bilateral networks of dorsal and medial prefrontal/frontal, as well as parietal activity. In the PMd/SM vicinity, additional bilateral contributions were observed more superiorly and medially (in SCEF, and 8BM), through mid- to anterior cingulate cortex (namely, a32pr, p32pr, and SFL, as well as 24dd, 24dv, and a24pr exclusively on the right). The superior parietal contributions for the left hand were also more extensive (and in addition to all parcels enlisted for the right hand, included IP2 and PFm on the left, and area 5mv, and 2 on the right). Yet, there were also additional bilateral clusters observed in frontal opercular regions (i.e., FOP5, FOP4, extending to area 44, 6r, and 6v on the right), and the bilateral anterior insular cortex (mainly AVI). Notably, in the right hemisphere, there was also an rMFG cluster invoked (involving area 46, p9-46v, a9-46v, and 9-46d). These effects are shown in Fig. 3F.

### Grasp execution

No significant differences between the networks involved in the execution of pre-planned pantomimes of the demanding grasp-to-use, easy grasp-to-use, or grasp-to-pass tasks were found for performance with the right hand. In the case of the left hand, executing pre-planned grasp-to-pass actions vs. demanding grasp-to-use actions revealed increased activity in bilateral visual cortices, left rMFG and left IPS. Visual cortices of the left hemisphere were also significantly more engaged in the execution of easy vs. demanding Grasp-to-Use actions. No other significant differences between grasp-related conditions of this study were found in the execution phase for the left hand.

### The results of MVPA

**Fig. 4:**
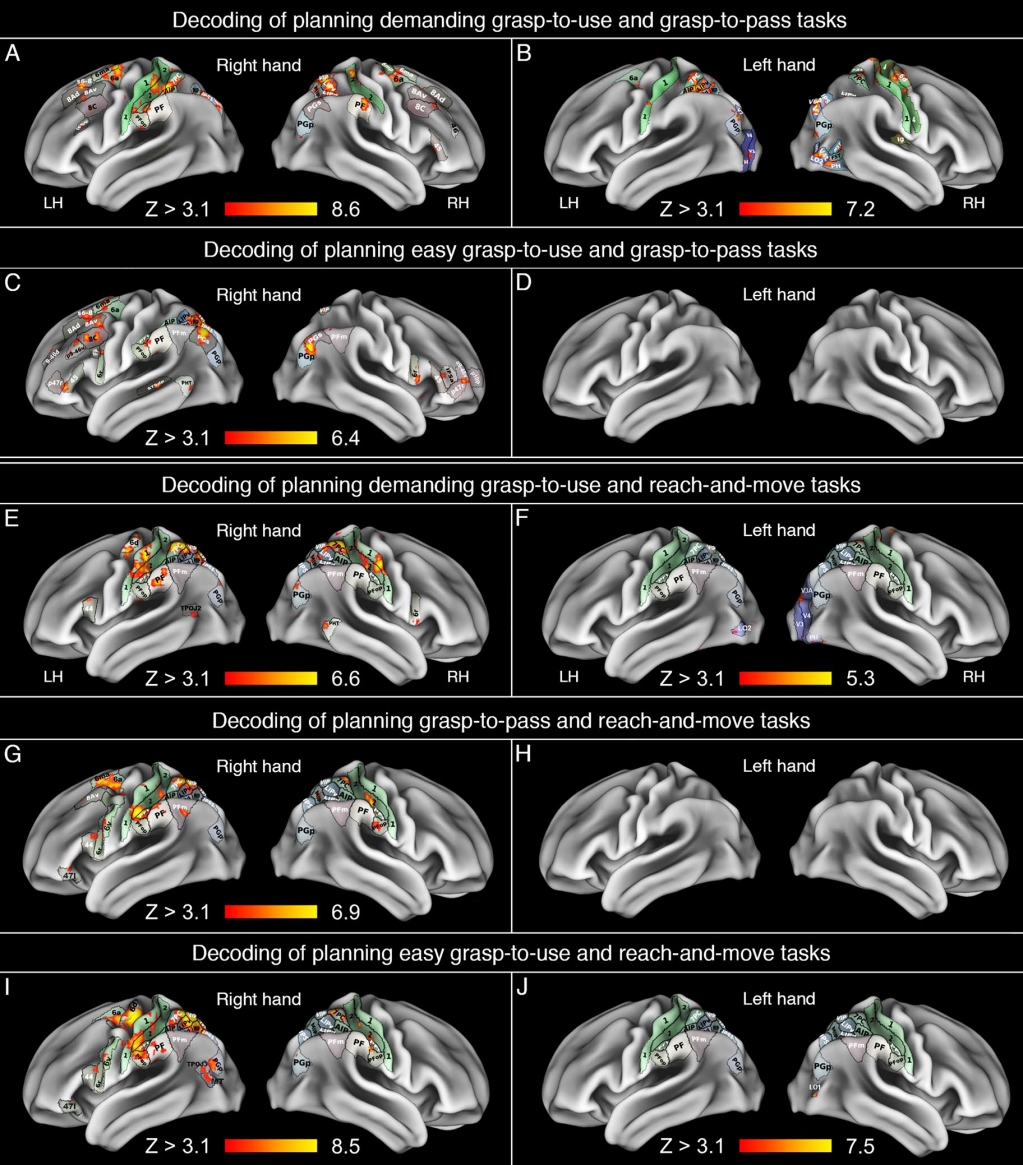
Brain areas showing significant decodings of planning grasp-to-use (GTU) and grasp-to-pass (GTP) tasks. **A** The demanding GTU (dGTU) and GTP tasks decoded for the right hand. **B** The dGTU and GTP tasks decoded for the left hand. **C** The easy GTU (eGTU) and GTP tasks decoded for the right hand. **D** The eGTU and GTP tasks decoded for the left hand. **E**–**J** The dGTU, GTP, and eGTU tasks decoded in the context of the reach-and-move (RAM) task, both for the right and left hand. Borders in panels **A**–**D** were displayed in areas where significant decoding accuracies were obtained. A constant set of parietal borders was used throughout panels **E**–**J** (except for cases with no decoding capabilities) as these borders correspond to the outcomes from the contrast involving planning reach-and-move action from the univariate analysis

Because MVPA was used here as a complementary method to the univariate analysis, the results below will be described in terms of similarities and disparities between the obtained outcomes. Decoding planning of dGTU and GTP tasks from fMRI signal with a searchlight procedure revealed all major fronto-parietal areas in both hemispheres as capable of discriminating between the two conditions. In the case of the right hand, the SPL clusters were typically located more ventrally, and the frontal clusters were more anterior (as compared to the ones revealed with the univariate analysis). Moreover, the searchlight analysis revealed numerous bilateral clusters capable of discriminating dGTU and GTP tasks in midIPS and the caudal intraparietal sulcus (cIPS), anterior IPL, and lateral prefrontal cortices. In both hemispheres, especially on the right, midIPS structures (such as MIP, but also LIPd and VIP), as well as more caudal subdivisions (IPS1, V6A, and V7) successfully decoded the two studied grasping conditions. So was AIP and its immediate vicinity on the left, and subdivisions of SMG bilaterally. While in the right hemisphere, the cluster within PF was located in the same vicinity as the subdivision showing greater activity for the dGTU task in the univariate analysis, the clusters in PFt and in its immediate vicinities on the left were revealed exclusively by MVPA. Significant decoding accuracies between the dGTU and GTP tasks were also observed in PMd, but were more anterior than the greater activity associated with planning dGTU revealed by the subtraction contrast. These outcomes and the remaining frontal and prefrontal clusters are shown in Fig. 4A.

As to decoding the two conditions for grasp planning performed with the left hand, left AIP and its immediate vicinities, as well as cIPS bilaterally, were successful in doing so in the left hemisphere. There were also numerous superior sensorimotor clusters involved in such decoding in the right hemisphere, as well as some limited involvement of left PMd (area 6a). The contributions from extrastriate and higher-order visual areas were also present, especially on the right. These effects are depicted in Fig. 4B.

Decoding planning eGTU and GTP tasks for the right hand, as shown in Fig. 4C, revealed only a subset of the fronto-parietal areas (e.g., with AIP, or 7PC missing) capable of discriminating the dGTU and GTP (cf. Fig. 4A). In IPS (starting from AIP, through its mid-subdivisions, as well as more medial cSPL vicinities), the ability to discriminate the two conditions was present, but not necessarily localized in the same manner as for the left hand in the dGTU and GTP tasks (Fig. 4B). Yet, it is of note that PFt/PF vicinity was invoked in this discrimination in a manner similar to the earlier decoding capability (Fig. 4A). Some consistencies (as compared to Fig. 4A) were also observed in lateral mid-to-superior prefrontal cortex. The most striking differences were detected in the inferior frontal cortices and in angular gyri bilaterally, as well as in the superior temporal sulcus (STSdp), and middle temporal gyrus (PHT) on the left. (Other more specific effects are also shown in Fig. 4C). No decoding discriminability was observed for the two tasks when performed with the left hand (Fig. 4D). Subsequent panels, i.e., E-J in Fig. 4, show decoding discriminability for the dGTU, GTP, and eGTU task in the context of the RAM task. Familiar sets of PRN areas were detected for the right hand (Fig. 4E, G, and I). Substantially weaker or no differentiation of these tasks was observed for the left hand (Fig. 4F, H, and J).

### The results of ROI analyses

**Fig. 5:**
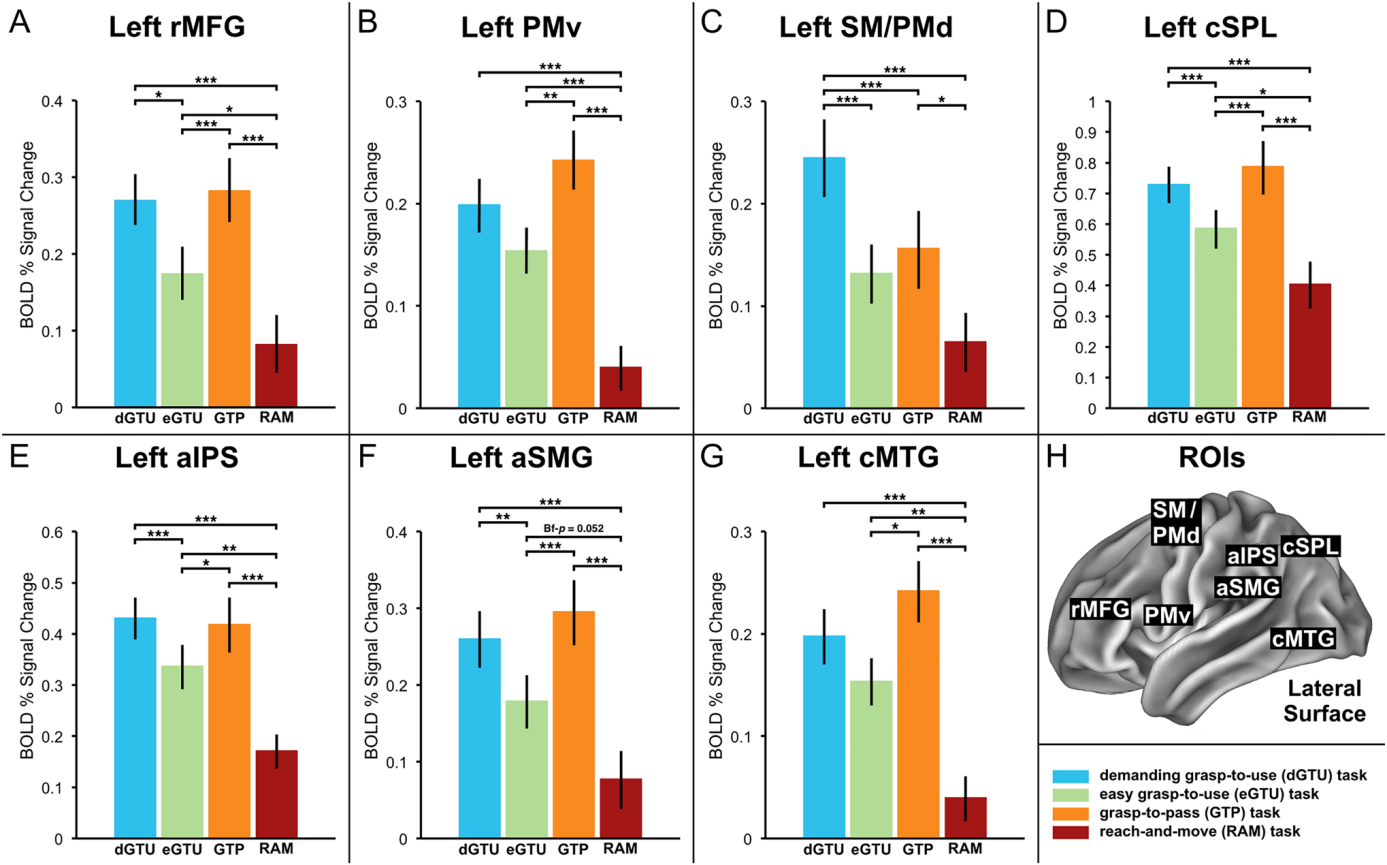
The results of ROI analyses for the planning phase. **A**–**G** Mean percent signal change within each ROI is plotted relative to the resting baseline for the following Tasks: demanding grasp-to-use (dGTU) task, easy grasp-to-use (eGTU) task, grasp-to-pass (GTP) task, and reach-and-move (RAM) task. (H) The overview of ROI locations depicted on the Connectome Workbench template brain. Asterisks indicate differences with Bonferroni-corrected *p* values of at least 0.05 (*), 0.01 (**), or 0.001 (***)

The results of 2 (Hand: right, left) × 2 (Phase: planning, execution) × 4 (Task: dGTU, eGTU, GTP, RAM) rmANOVAs calculated separately for each of the selected ROIs are presented in Table 1. Only SM/PMd demonstrated a significant main effect of Hand such that performing tasks with the right hand was associated with greater activity than performing tasks with the left hand. All of the remaining ROIs were invoked to a similar extent by both hands. PMv, SM/PMd, aIPS, and aSMG showed a significant main effect of Phase such that action planning engaged these areas more than action execution, whereas the opposite effect was found exclusively in cMTG. A significant main effect of Task was observed in all ROIs, and in each case, it was accompanied by a significant Phase by Task interaction. Exploring these interactions revealed that in the majority of ROIs the main effects of Task were driven primarily by significantly different levels of activity associated with planning disparate actions, and not their execution. No significant three-way interaction was detected. The outcomes of Phase by Task interactions are presented in detail below, and the planning-related differences between Tasks in each ROI are displayed in Fig. 5.

In five ROIs — i.e., rMFG, PMv, aIPS, cSPL, and cMTG — the activity related to planning each of the grasping tasks exceeded the one for planning the RAM task. In aSMG, the difference between planning eGTU and RAM tasks just missed the adopted significance level (Bf-*p* = 0.052), while in SM/PMd, there was no significant difference between the two tasks (Bf-*p* = 0.16). Yet, SM/PMd was the only area that demonstrated significantly greater engagement for planning dGTU than GTP task. This outcome is presented in Fig. 5C. rMFG, cSPL, aIPS, and aSMG were invoked more for planning both dGTU and GTP tasks than eGTU task (see Fig. 5A, D, E, and F, respectively). PMv and cMTG were engaged more in planning GTP than eGTU tasks, but demonstrated no significant difference between planning dGTU and eGTU tasks (see Fig. 5B and G, respectively). Conversely, SM/PMd was invoked more for planning dGTU than eGTU tasks, but did not differentiate between planning GTP and eGTU tasks.

During the execution phase, aIPS was significantly more engaged by the GTP task than any of the other tested tasks (Bf-*p* < 0.05 in each case), whereas cSPL was invoked more by the GTP than eGTU task (Bf-*p* < 0.05). In SM/PMd, execution of the RAM task was associated with significantly stronger activity than the eGTU task (Bf-*p* < 0.001) and GTP task, and executing the dGTU task was linked to higher activity than eGTU task (Bf-*p* < 0.05).

A significant Hand-by-Phase interaction effect was found in aSMG and rMFG. The analysis of simple main effects revealed that in both cases, planning actions with the right hand was associated with greater activity than planning actions with the left hand, although only in the case of rMFG did the effect reach the Bonferroni-corrected significance level (aSMG: *p* = 0.059; rMFG: *p* < 0.01).

## Discussion

Because action goals and the associated intentions can be represented in the brain in the absence of overt movements, prior to or even without real interactions with objects (e.g., Tunik et al. 2008; Gallivan et al. 2011; Malfatti and Turella 2021), and pantomimed actions are a valid proxy to studying neural circuits of real actions (Króliczak et al. 2007; Garcea and Buxbaum 2019; see also Sulpizio et al. 2020), here, using fMRI, we tested the neural underpinnings of planning and pantomimed execution of disparate grasps of tools with different goals in mind. Consistent with our main hypothesis, the obtained results show that regardless of the used hand, all major components of left-lateralized PRN were engaged in planning both functional and non-functional/structural grasps of tools more than in reaching actions with an intention to move or push tools as obstacles. Yet, in contrasts with the latter task, there were also significant contributions from the right hemisphere counterparts of the identified PRN nodes. This is the case because the planning of disparate grasps was performed on stimuli which — for different goals — would also necessitate quite complex cognitive control, involving attentional, visuo-spatial, and even “social” mechanisms (e.g., Gotts et al. 2013), in addition to the requisite visuo-motor processing. Nevertheless, the demanding and easy functional grasps, when directly contrasted with the convenient structural grasps, modulated the PRN activity in different, and even unexpected manners.

In accordance with earlier proposals (e.g., Binkofski and Buxbaum 2013), preparation of exclusively demanding functional grasps, when compared to rather convenient structural grasps to pass, invoked primarily the superior, dorso-dorsal streams of processing (the *grasp* system; Rizzolatti and Matelli 2003; Goodale et al. 2005), typically associated with increased requirements for sensory–motor mechanisms contingent on achieving disparate goals. Importantly, because the lateralization of the dorso-dorsal processing was here mainly hand-dependent, i.e., except for right PMd (and SMG), it was left-lateralized for the right, and right-lateralized for the left hand (wherein it also included some medio-dorsal contributions), it could be linked to lower-level processing of tool orientation for the required grasping hand postures. Consistent with this notion is the observation that such differences in neural activity did not emerge when merely easy functional grasps were compared to convenient structural grasps. While the orientations of tools differed in the latter comparison, they were irrelevant for the required grasps. It should be also noted that having tool stimuli in both of the contrasted tasks, which are matched for initial movement complexity, would result in subtraction of some of the higher-order processing of the to-be-grasped tools. E.g., the initial retrieval of conceptual tool knowledge is expected to take place in both of the compared tasks, and therefore would be removed from the outcome of such a comparison.

Critically, counter to a naïve hypothesis that PRN mediates only tool use actions, but consistent with a notion that it also encodes “the intentions and goals of the actor, and interpretations of prevailing task demands” (Kroliczak and Frey 2009, p. 2408), planning structural grasps to pass engaged some subdivisions of the left ventro-dorsal processing stream (the *use* system; and even the dorso-dorsal nodes of the *grasp* system; Rizzolatti and Matelli 2003; Frey 2007) more than functional grasps, but only when compared to the easy grasps. Because the obtained activity was then exclusively left-lateralized, regardless of the used hand (and purely hand-dependent outcomes are typically contralateral), it calls for a higher-order “interpretation” of this particular result. In our view, the greater engagement of the rostral middle frontal cortex for planning grasps to pass can be related to processing of a wider action context.

As to further contrasts and their results, consistent with the idea that bilateral parieto-frontal circuits control sensory-motor transformations underlying *acting on* objects (Johnson and Grafton 2003), including functional interactions with tools contingent on their orientations (Styrkowiec et al. 2019), demanding vs. easy functional grasps of tools invoked the dorso-dorsal streams even more than in a comparison with planning structural grasps to pass. Yet, an interpretation of this effect can be limited mainly to lower-level processing, i.e., differences in the requisite hand orientations for actions having exactly the same goal. Last but not least, similarly to earlier research using MVPA (Malfatti and Turella 2021), our report also shows that numerous bilateral parieto-frontal areas can accurately decode disparate interactions with tools, be it functional or not, with some differences contingent both on action intentions and target stimulus properties. These outcomes suggest that the PRN contribution to planning functional and structural grasps of tools is modulated by higher-order cognitive factors involved in processing disparate action goals. Yet, depending on the tasks being compared, complementary right hemisphere processing is revealed (Gotts et al. 2013; Vingerhoets 2019), given essential sensorimotor demands imposed by the tool-actor, and actor-actor relationships, including their egocentric positioning and relevant hand-, arm- or even body-related frames of reference.

### PRN and representations of disparate action goals

The engagement of the critical PRN areas in performance of functional grasps (limited to nodes from the ventral processing stream and their right hemisphere counterparts; Fig. 2A), and tool use pantomimes (the whole network in Fig. 2B), as well as planning both functional and non-functional grasps of tools (e.g., Fig. 2CD; and Fig. 3CD see also Fig. 4), regardless of action goal, is consistent with the outcomes from numerous fMRI studies. The latter include research on pantomimed tool use gestures (Johnson-Frey et al. 2005; Fridman et al. 2006; Bohlhalter et al. 2009; Kroliczak and Frey 2009; Vingerhoets et al. 2011), planning functional grasps of tools (Przybylski and Kroliczak 2017; Styrkowiec et al. 2019; Knights et al. 2021), and performance of familiar actions involving tools (Valyear et al. 2012), including tool–recipient interactions (Brandi et al. 2014). The outcomes observed in our main study, after subtracting brain activity invoked by the *reach-to-move* or push actions (cf. Króliczak et al. 2008), were virtually identical regardless of the hand used in grasp performance, which also points to the engagement of some higher-order mechanisms for the control of these skills. The notable additional involvement of the right hemisphere counterparts of numerous PRN nodes revealed in this contrast is unlikely to be specific for interactions with tools. Instead, it can be linked to greater demands for attentional processes, egocentric perspective taking, as well as visuo-spatial transformations (Astafiev et al. 2006; Króliczak et al. 2007; see also Rossit et al. 2011).

The striking differences observed in anterior SPL, sensorimotor, and dorsal premotor regions revealed by the comparisons between planning demanding functional grasps and planning structural grasps (Fig. 3AB; cf. Figure 4AB), are not only consistent with effects reported in earlier studies on selection of stimulus guided movements (e.g., Grafton et al. 1998; see also Fabbri et al. 2014; Macuga and Frey 2014), but also associate this neural activity with increased requirements imposed by greater kinematic demands for functional grasps, including prerequisite hand rotations (cf. Marangon et al. 2011). This effect was, however, more hand-dependent and can be also linked to lower-level or simple visuo-motor processing. It might be worth mentioning, though, that right aSMG (PF), a subdivision previously linked to grasping complex objects (Marangon et al. 2016), was more involved in the planning of demanding functional grasps with the right hand. This observation is consistent with the reported right aSMG contribution to selection/modification of appropriate hand/finger orientations while grasping tools (McDowell et al. 2018; Ras et al. 2022; see also Potok et al. 2019).

Partially counter to one of our hypotheses emphasizing the greater role of PRN in functional interactions with tools, and in contrast to an earlier research showing no comparable differences in the left hemisphere for pantomimed tool transport relative to tool use actions (Garcea and Buxbaum 2019), our main study shows that planning convenient structural grasps, with a view to passing a tool to a different person, invoked the left rMFG and SPL, and to some extent also IPL/IPS (though less for the left hand, Fig. 3CD), substantially more than planning easy functional grasps. These outcomes are also inconsistent with findings from our background localizer on the simpler grasp-to-displace task involving tools (Fig. 2A), which revealed greater engagement of the right hemisphere IPL, that can be linked in this context to flexible reconfiguration of tool grasping behavior (Singh-Curry and Husain 2009). Namely, in this localizer task, tools were grasped to be moved away, not to be used, and the right parietal activity can be associated with different visuo-spatial processing required for achieving such a goal. Notably, the easy functional grasps in the main experiment were planned with a view to using, but without pantomimed use itself, and were performed on differently, yet more intuitively oriented tools. Such orientations naturally invoked grip kinematics similar to the grasp-to-pass condition, but in contrast to the latter did not require any reconfiguration of grasping behavior. Hence, the differences between their neural underpinnings were observed elsewhere, i.e., in prefrontal and superior parietal regions. (For completeness, see also Fig. 4AB showing bilateral decoding capabilities for the two tasks, and even between easy functional grasps and grasps to pass, Fig. 4C; unfortunately, these outcomes cannot be directly linked to any greater engagement of an area revealed by a univariate analysis). Only two regions — left rMFG and SPL — are emphasized while discussing this effect because their greater roles in structural grasp planning were again observed independently of the hand, whereas contributions from supplementary and premotor areas were inconsistent (at least in terms of location), and in the case of the SMG (PF)/IPS (AIP) vicinity nearly absent for the left hand. Indeed, consistent with the weaker effect for the non-dominant hand, MVPA revealed less (Fig. 4B) or even no decoding efficiency (Fig. 4D) for discriminating between functional and structural grasps when the left hand was used in grasp planning. Univariate ROI analyses indicated, however, that most of the tested areas were more involved in planning structural vs. easy functional grasps, too (Fig. 5AB, D–G). These same analyses, partly inconsistent with the outcomes from the (easy) grasp-to-displace localizer task (Fig. 2A), further suggest that cMTG is also recruited for non-functional actions on tools. This region most likely represents all possible interactions with target tools (e.g., Watson and Buxbaum 2015), and the repertoire of possible interactions in tool-directed tasks is then substantially narrowed by SMG just before object manipulation, contingent on action context (Boronat et al. 2005).

Apparently, the contribution of PRN to the control of easy functional grasp is quite automatic, or does not require any complex operations on conceptual and visual (structural) inputs, given their standard positioning, including their orientations with respect to the acting hand. Indeed, the planning of easy functional grasps invokes quite standard, and automatic grasping responses required when such tools are needed in action. Structural grasps to pass, conversely, necessitate that the natural functional response (an appropriate hand rotation) is abandoned, given the action goal and its modified target, being now a different person, rather than a recipient object to be acted on.

The greater bilateral engagement of the superior, dorso-dorsal parieto-frontal pathways for more demanding (vs. easy) functional grasps (Fig. 3EF) is again consistent with the notion that these processing streams are involved primarily in the control of biomechanical demands imposed on the acting hand (e.g., Goodale et al. 2005; Sakreida et al. 2016; see also Gamberini et al. 2020; Sulpizio et al. 2020; Tosoni et al. 2015), even when actions are performed on tools (Styrkowiec et al. 2019). In other words, the bilateral superior parieto-frontal circuits revealed in these contrasts most likely contribute to sequencing of sensory-motor transformations while *actions on* target tools are planned, rather than to the processing of tool functions and tool-related skills (Johnson and Grafton 2003). In agreement with earlier accounts (Davare et al. 2006; Króliczak et al. 2008; Monaco et al. 2011; Begliomini et al. 2015), both PMd and several SPL areas were invoked more for planning the demanding functional grasps, but now also regardless of the hand. Yet, given that the responses were directed at the same target tools and triggered by the same action goal, these differences cannot be attributed to higher-order processing. The only unexpected effect was the bilateral contribution from the medio-dorsal processing stream (including SMA), as well as posterior IFC, anterior insula and right rMFG, exclusive for planning demanding functional grasps with the left hand (Fig. 3F). Perhaps the more deliberate performance of demanding functional grasps with the non-dominant hand is more computationally challenging. Therefore, bilateral contributions from the insula — a hub linking disparate action systems, including the nearby frontal and prefrontal structures — are critical here, as well (Kurth et al. 2010; Bidula and Kroliczak 2015; Kroliczak et al. 2016).

While some of the outcomes from the MVPA analysis seem to corroborate the left hemisphere advantage for decoding capabilities between tool-directed grasps and reaches (Fig. 4, e.g., subdivision PF in panel E, see also panels G and I), the remaining ones show rather bilateral contributions to such decoding. Even more surprisingly, this is only the case for the dominant right hand. Apparently, the non-dominant hand (see Fig. 4, panels F, H, and J) has no privileged access to these decoding capabilities.

The lack of significant between-task differences in the PRN for pantomimed grasp execution for the right hand is somewhat disappointing, too. Yet, these outcomes are similar to the ones observed elsewhere (Przybylski and Kroliczak 2017). There were no substantial differences in the grasp execution phase, either. Some weak effects for the left hand were present, e.g., in left rMFG and IPS for the GTP vs. dGTU task, and they were equally surprising to us as the differences observed in the preceding planning phase. The sustained processing of wider action context, and the use of the less skilled hand must have contributed to this effect.

Finally, an interesting spinoff emerged from our tool use localizer task. Not only it corroborated that the control of tool use pantomimes is strongly left-lateralized (e.g., Frey 2008) but it also showed that the control of simulated animal movements, in response to pictures of animals is strongly right-lateralized in the fusiform cortex, and posterior precuneus (Fig. 2B). On the other hand, the contributions from early visual cortices that were also involved more in this task (as compared to pantomimed tool use) were clearly more balanced or bilateral. Unlike the unilateral engagement of the precuneus, often linked to visuo-spatial imagery (Cavanna and Trimble 2006), but also to generating animal names (Vitali et al. 2005), the bilateral involvement of lower-level visual areas is not surprising because they rarely show lateralized activity (here, except for V4t, and MT) for processing objects that occupy both visual fields. The right-lateralized contribution from higher-order processing in the fusiform cortex is, however, consistent with a recent report from a patient study (Henderson et al. 2021), showing category-selective semantic deficits for matching pictures and words denoting animals, following cortical atrophies involving the fusiform gyrus. In agreement with our findings, the effect was associated with larger (in their extent) atrophies located in the right hemisphere ventral temporal cortices, including anterior fusiform gyrus (and substantially smaller atrophies limited only to the left anterior fusiform cortex).

### PRN, disparate action goals and affordances

The more skilled, stereotyped and/or natural a tool-related action is, the more likely it is to be associated with weaker brain activity (e.g., Kroliczak and Frey 2009). If certain functional tool features, the so-called *affordances* (Gibson 1977; Mizelle et al. 2013; Kourtis and Vingerhoets 2015; Michałowski and Króliczak 2015; Belardinelli et al. 2016; Federico and Brandimonte 2020; for recent reviews, see Osiurak et al. 2017, 2020) automatically potentiate such skilled actions as functional grasps, then the planning of grasp-to-pass action would require greater effort. Thus, in accordance with the encoding specificity principle (Tulving and Thomson 1973), retrieval of representations of tools and their standard use in response to images of tools, the more so real objects, should be much easier than retrieval and organization of actions necessary to pass an object to someone else (Osiurak et al. 2013). Indeed, the latter seems to require a substantial change in motor strategy when embedded among more automatic interactions with tools (such as their use) and/or less demanding tasks (such as pushing tools away), wherein the typical tool-related brain responses have to be inhibited (Vainio and Ellis 2020), and there is greater burden on working memory (Pilacinski et al. 2020). Perhaps the pattern of PRN responses would be different if the change in motor strategy was more predictable or not required at all (e.g., Valyear et al. 2011). Although there are studies raising doubts about automaticity of affordance effects and suggest that grasp responses emerge from goals rather than from the affordance compatibility with the presented tools, we cannot fully exclude the interference involved in the retrieval of typical, memory-based action representations (Bub et al. 2021; Masson-Carro et al. 2020).

Alternatively, planning of grasp-to-pass actions may naturally invoke thoughts/images of a potential receiver or even interactions with him/her. This more deliberate approach, potentially invoking thoughts of complex prospective interactions would be also expected to engage the brain more (but see Foerster et al. 2020). In other words, although not inconsistent with the notion that PRN is particularly sensitive to constant functional properties of objects or *stable affordances* defining tool functionality (Borghi and Riggio 2015; Sakreida et al. 2016), the greater engagement of the ventro-dorsal PRN can be also linked to the control of intended actions in more complex cognitive settings or contexts. Yet, this idea still corroborates that PRN is a versatile neural system whose task is to dynamically integrate and transform both conceptual knowledge (e.g., in LOTC, Lingnau and Downing 2015; or cMTG complex, Kubiak and Króliczak 2016), the planned, prospective and/or real sensorimotor computations (cSPL, aSMG/aIPS, PMv) and contextual inputs (rMFG) into purposeful acts or praxis skills (Frey 2008; Kroliczak and Frey 2009; see also Riccardi et al. 2020). All these regions have been shown, both here and previously, to be capable of decoding disparate action goals, and even general functions of tools by which these goals are achieved (Malfatti and Turella 2021), though sometimes in different action phases. The dorso-dorsal nodes of PRN are clearly involved in fast, and automatic, perhaps mainly on-line processing of object structure, position, and orientation for the grasping/acting hand, and are less concerned with detailed processing of functional affordances as such. Nevertheless, a rough information on object function must be also provided to these regions, most likely by rMFG and/or cMTG (Goodale et al. 2005).

The apparent contradiction between the outcomes of a study by Garcea and Buxbaum (2019) and ours can be easily explained by the nature of tasks performed in these disparate projects. In our study participants only planned the grasp with a view to using or moving a tool, whereas in the study by Garcea and Buxbaum, they planned and executed tool use or tool transport actions, the tasks which inevitably change the dynamics of processing within critical PRN nodes. Future studies should aim at combining the disparate phases, rather than studying them in isolation (Macdonald and Culham 2015; Potok et al. 2019; Styrkowiec et al. 2019; Matic et al. 2020; Monaco et al. 2020) to investigate the dynamic changes or signal modulations within PRN during the full sequence of action steps.

### Limitations of our study

One of the potential limitations is a lack of the demanding structural grasp-to-pass condition. Yet, at least in terms of the required movement kinematics it would be, by definition, much harder than an easy functional grasp-to-use action. Therefore, in addition to the already observed differences in the ventro-dorsal stream, it should lead to the greater engagement of the dorso-dorsal stream, as well. Moreover, a justification would be needed why a participant should incorporate hand rotation in grasp planning, instead of using the most spontaneous grasp for the control object orientations. Conversely, if we included control orientations for the easy structural grasps, instead, it would not only result in a more asymmetric design, wherein only a subset of trials (for the demanding functional grasp, only) would require substantial hand rotations, but could also put it into question why disparate object orientations (or alternatively, demanding hand rotations) were not included for the reach-to-move actions. Finally, although the use of pantomime is well established in neuroimaging studies of praxis skills, especially the ones concerned with the planning processes, any differences in the outcomes of real and pantomimed grasping of tools and/or subsequent actions with tools, be it functional or not, can be revealed only when they are simultaneously studied (Hermsdorfer et al. 2007; Króliczak et al. 2007; see also Kithu et al. 2019; Whitwell et al. 2020). Future research could/should also address representations of disparate action goals in other handedness groups, and/or in individuals with atypically represented praxis skills (Kroliczak et al. 2021).

### Main differences between our study and earlier research on tool use skills

Our project examined modulations of neural activity contingent on performance of disparate functional and non-functional grasps of tools (i.e., actions preceding tool use or other types of tool handling, e.g., passing them), based on pictorial cues — here, high-resolution visual images of tools. In traditional neuropsychological paradigms revealing greater contributions of the left hemisphere to praxis skills, patients/participants are asked to pantomime tool use gestures to verbal commands, or displayed linguistic stimuli. Such tests enforce the retrieval of stored tool concepts and action representations based on minimally informative cues (Liepmann 1900; Goldenberg 2003; Kroliczak and Frey 2009). Moreover, counter to neuropsychological research, wherein tool use deficiencies are assessed based on action performance, we observed between-task differences mainly in the planning, not execution phases of the studied tasks. Furthermore, in our main study, tool pictures served as stimuli in all conditions. Therefore, it can be assumed that the initial retrieval of conceptual tool knowledge was virtually the same in each task. Indeed, the main between-task differences were the selection of grasping hand postures, and/or the intended recipients or action goals. Meanwhile, the relevant neuropsychological literature focused on differences between the neural underpinnings of grasping and using tools (e.g., Randerath et al. 2010). To the best of our knowledge, in the only neuropsychological report which studied an issue similar to ours, the effects of conflict imposed by grasp selection mechanisms — for *using* tools vs. *moving* them, based on pictorial cues — were again assessed only in the accuracy of tool use pantomimes (and tool recognition), not in actions involving moving objects away or to the side (Watson and Buxbaum 2015). Not surprisingly, this study also identified left-lateralized neural underpinnings for pantomimed tool use (which was more affected in the case of the so-called conflict objects). Finally, it should be emphasized that whether or not left-lateralized activity for tool-related actions is revealed in fMRI research will critically depend on the characteristics of the control task, i.e., whether tools or other objects are used, and how complex the control task performed on them really is. Indeed, we have further evidence that in contrast to planning and execution of tool use gestures (pantomimes), engaging primarily the left hemisphere, real grasping movements and later use of more complex tools are associated with more balanced neural activity and, depending on a specific contrast, can be linked to even greater contributions of the right hemisphere, too (Ras et al. 2022).

## Conclusion

While the present results are still consistent with an idea that the left-lateralized temporo-parieto-frontal praxis network represents quite disparate types of interactions with tools, our study also shows when, and why greater right hemisphere contributions are required during such interactions. We demonstrated these effects for such basic action prerequisites as planning tool-directed grasps, regardless of whether they are functional or structural. Unexpectedly, the latter can engage prefrontal and parietal subdivisions of PRN more, but only when compared to simple grasp-to-use. Thus, our research shows that PRN is differently modulated by such tasks and its contribution, regardless of the utilized hand, depends on movement complexity, directly related to tool orientation, and indirectly to the kind of intended actions, the associated cognitive settings, and even broader action contexts. An important question for future research is to investigate disparate phases of actions involving tools, such as preparation for grasping, grasping itself, and later use or displacement, in more ecologically valid action settings.

## Data Availability

De-identified data are available upon reasonable request from the corresponding author.

## Appendix

A list of tools used in the main experiment: clothes peg, comb, dropper, eraser, hammer, key, nail file, rake, screwdriver, spatula, razor, wrench.

A list of tools used in the Tool Use Localizer: ax, hammer, knife, masher, paintbrush, peeler, pliers, screwdriver, shovel, scissors, wrench.

A list of animals used in the Tool Use Localizer: bat, bear, dolphin, elephant, fish, horse, hummingbird, orca, parrot, pigeon, sea turtle, wolf.

A list of tools used in the pantomimed structure-based Grasp-to-displace (or move) Localizer: corkscrew, lighter, scissors, sprinkler, syringe, toothbrush.
